# Finding new ways to treat overdoses

**DOI:** 10.7554/eLife.105511

**Published:** 2025-01-27

**Authors:** Jill R Turner, Jocelyn Martin

**Affiliations:** 1 Department of Pharmaceutical Sciences, University of Kentucky Lexington United States

**Keywords:** opioids, fentanyl, overdose, naloxone, naloxone methiodide, withdrawal, Rat

## Abstract

Reversing opioid overdoses in rats using a drug that does not enter the brain prevents the sudden and severe withdrawal symptoms associated with therapeutics that target the central nervous system.

**Related research article** Ruyle BC, Sarah M, Rohith K, Mubariz T, Juhi M, Caroline R, Sofia AL, Tania L, Higginbotham Jessica A, Nicolas M, Moron Jose A. 2024. Peripheral opioid receptor antagonism alleviates fentanyl-induced cardiorespiratory depression and is devoid of aversive effects. *eLife* **13**:RP104469. doi: 10.7554/eLife.104469.

While the societal and human cost of opioid use disorder is now widely recognized, there are still few evidence-based interventions to treat those experiencing addiction, including strategies for the prevention of overdoses. The gold standard rescue agent for opioid overdose remains naloxone – a life-saving drug now available over the counter (FDA, 2023), which works by binding to mu opioid receptors (known as MORs) in the brain and abruptly disrupting their signaling.

However, those treated with naloxone often report that being rescued from an overdose was a highly traumatic, painful experience. The treatment triggers immediate and severe withdrawal symptoms, including vomiting, diarrhea, abdominal, bone and muscle pain, altered consciousness, elevated heart rate, and agitation (Hassanian-Moghaddam et al., 2014; Lai et al., 2021).

MORs are found across the central nervous system and are highly enriched in certain brain areas (Le Merrer et al., 2009) that are known to control multiple aspects of opioid overdose, such as respiratory depression (Vázquez-León et al., 2021; Aicher et al., 2000). This decrease in, or complete loss of, breathing is the leading cause of opioid-related death (Zedler et al., 2018).

However, physical access to MORs in the brain is regulated by the blood-brain barrier – a selective, semi-permeable membrane that separates the central nervous system from the rest of the body (Ballabh et al., 2004). Although naloxone can cross this barrier, many compounds cannot, and this has been a key stumbling block for drug development (Pardridge, 2005). Now, in eLife, Jose Moron and colleagues at Washington University in St. Louis – including Brian Ruyle as first author – report that blocking MORs outside the brain can reverse opioid overdoses in rats without inducing the severe withdrawal symptoms associated with naloxone (Ruyle et al., 2024).

First, Ruyle et al. investigated the precise mechanisms underlying opioid-induced respiratory depression. Administering the synthetic opioid fentanyl to rats lowered their oxygen saturation measurements, confirming that it induces respiratory depression (Figure 1A). The experiments also revealed that fentanyl increases the activity of neurons in an area of the brain called the nucleus of the solitary tract, in a dose-dependent manner. This area contains many MORs and heavily regulates breathing as it is the first to place to receive sensory information about oxygen levels (Baptista et al., 2005; Aicher et al., 2000; Zhuang et al., 2017).

**Figure 1.**
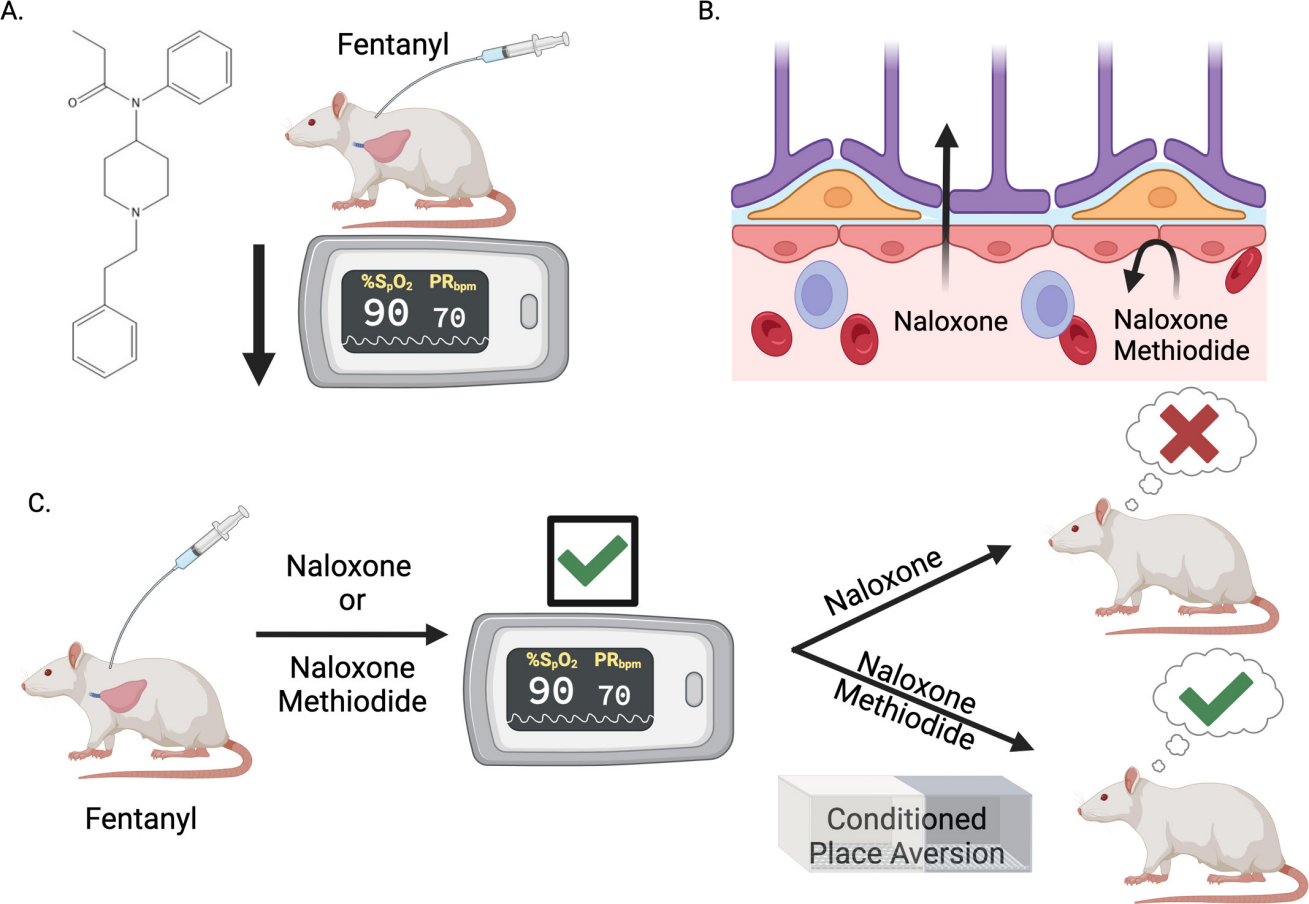
Blocking opioid receptors outside the brain reverses opioid overdose without sudden and severe withdrawal symptoms. (**A**) The synthetic opioid fentanyl can inhibit breathing (known as respiratory depression) in people who use the drug. Experiments by Ruyle et al. that measured blood oxygen levels confirmed that fentanyl has the same effect on rats. (**B**) The gold standard medicine for treating opioid overdoses is naloxone (left), a drug that crosses the blood-brain barrier and binds to opioid receptors in the central nervous system. This rescues respiratory depression but leads to sudden and severe withdrawal symptoms, which patients report as traumatic. Ruyle et al. explored whether an alternative compound called naloxone methiodide (right), which binds to opioid receptors but cannot cross the blood-brain barrier, can rescue respiratory depression without inducing the negative side effects associated with naloxone. (**C**) To test this, Ruyle et al. treated rats that had been administered fentanyl with either naloxone or naloxone methiodide. Both treatments successfully reversed the fentanyl-induced respiratory depression. A conditioned place aversion experiment was used to test whether either of the treatments caused distressing symptoms. Rats treated with naloxone showed aversion to the compartment in which they were treated, whereas rats treated with naloxone-methiodide did not display aversion. This suggests that naloxone-methiodide can rescue respiratory depression without the negative side effects induced by naloxone, representing a potential new therapeutic avenue to explore in humans. This figure was created with BioRender.com.

With this knowledge, Ruyle et al. next assessed whether an MOR antagonist called naloxone-methiodide (which is derived from naloxone but does not cross the blood-brain barrier) could rescue fentanyl-induced respiratory depression (Figure 1B). Treating rats with naloxone-methiodide either before or after administering fentanyl successfully prevented or reversed respiratory depression, respectively. This approach of blocking MORs outside the brain was as effective as naloxone treatment, without the requirement of crossing the blood-brain barrier (Figure 1C). In tandem, Ruyle et al. also used immunofluorescence and a technique called fiber photometry to demonstrate that blocking peripheral opioid receptors affected the fentanyl-induced activity of the nucleus of the solitary tract neurons, revealing a mechanism behind naloxone-methiodide’s action.

The most striking finding described by Ruyle et al. came when they used an approach called ‘conditioned place aversion’ to assess if blocking peripheral MORs reduces the severity of the withdrawal symptoms. In this procedure, rats were given fentanyl in one compartment and then treated with naloxone or naloxone-methiodide in another. Afterwards, the rats treated with naloxone showed aversion to the compartment in which they had been treated, suggesting that their experience had been distressing. However, those treated with naloxone-methiodide did not exhibit this behavior, suggesting that the treatment, while effective as a respiratory depression rescue agent, also did not cause unpleasant withdrawal symptoms (Figure 1C).

Taken together, the findings highlight a new therapeutic avenue to address opioid overdose without eliciting distressing withdrawal symptoms. This approach could be highly beneficial for harm reduction in opioid use disorder, given that many clinical reports show that the effects of naloxone-mediated withdrawal can lead to a reluctance to administer it in overdose scenarios (Hall et al., 2024). Furthermore, concerns about treatment-induced withdrawal symptoms are often a reason for individuals not seeking other evidence-based treatment for opioid use disorder (Spadaro et al., 2022; Kosten and Baxter, 2019). This important work by Ruyle et al. reframes our understanding of successful overdose rescue and could lead to future treatments with reduced withdrawal effects.
